# Diameter-Dependent Carbon Nanotube Hydrogel Formed with Tannic Acid and Its Application in Thermoelectric Power Generation

**DOI:** 10.3390/nano15201556

**Published:** 2025-10-13

**Authors:** Nobuyasu Okubo, Takahide Oya

**Affiliations:** 1 Graduate School of Engineering Science, Yokohama National University, Yokohama 240-8501, Japan; 2 Semiconductor and Quantum Integrated Electronics Research Center, Institute for Multidisciplinary Sciences, Yokohama National University, Yokohama 240-8501, Japan

**Keywords:** carbon nanotube, carbon nanotube dispersion, hydrogel, dispersant, conductive gel, thermoelectric power generation

## Abstract

In this study, we discovered a new diameter-dependent carbon nanotube (CNT) hydrogel composed exclusively of CNTs and tannic acid (TA). Accordingly, we first examined the relationship between the concentrations of CNTs and TA, as well as the CNT diameter, and whether gelation occurred. As a result, we found that when the TA concentration was fixed at 0.15 wt%, the threshold CNT concentration required for gelation was 0.05 wt%, which was lower than the values reported for previously known CNT hydrogels. We also determined that a TA to CNT weight ratio of 2–3 is critical for gelation. Furthermore, we found that subjecting the CNT dispersion to hydrothermal treatment at 160 °C, followed by freezing and ambient drying, produced a CNT aerogel that retained its 3D structure. Then, we evaluated the thermoelectric properties (electrical conductivity and Seebeck coefficient) of the resulting CNT hydrogel and aerogel under a temperature gradient for application. Both materials exhibited stable and reproducible electromotive force, and the measured Seebeck coefficients were comparable to those of conventional CNT-based thermoelectric materials. These findings demonstrate that 3D thermoelectric materials can be readily fabricated from CNT dispersions via simple processes and highlight the potential of these materials for future applications in energy-harvesting devices.

## 1. Introduction

**Figure 1:**
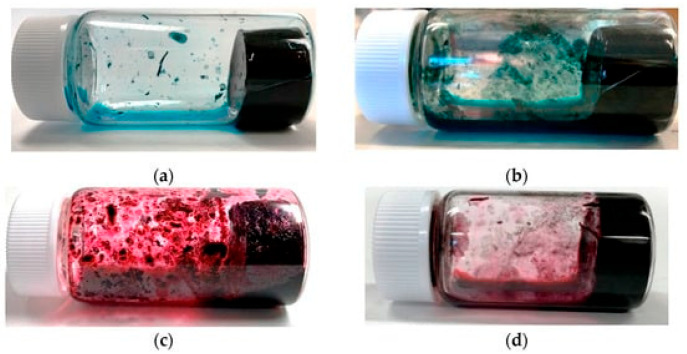
CNT hydrogels produced from a combination of the following: (**a**) (6,5)-chirality CNTs and C.I. Reactive Blue 21; (**b**) CG300 and C.I. Reactive Blue 21; (**c**) (6,5)-chirality CNTs and Cyanocobalamin; (**d**) CG300 and Cyanocobalamin. (From Ref. [15] under license CC BY 4.0.).

It is known that carbon nanotubes (CNTs) [1] have many useful characteristics, such as high electrical conductivity, high mechanical strength, and thermal conductivity. In addition, their electrical properties can be either metallic or semiconducting depending on their structure [2,3,4,5]. However, CNTs tend to aggregate unevenly due to intermolecular forces such as π–π interactions, resulting in the formation of bundled structures [6]. It has been found that, in this aggregated state, CNTs are unable to display some of the above characteristics that they originally possessed [7,8]. Therefore, CNTs are difficult to handle for their use and application, and many efforts have been made to handle them. For applications using CNTs, many efforts have been made to develop composite materials combining CNTs with other materials and, actually, the development of many CNT composite materials and applications using them have been reported [9,10,11,12,13,14]. On the other hand, as is common in all research, efforts have been made to disperse and stabilize them in water before incorporating them into composite materials. In other words, how to prepare CNT dispersions is very important for the application of CNTs. In our previous studies, we explored a variety of dispersants for the development of CNT applications. In the exploration, we found that CNT dispersions made with certain combinations of CNTs and dispersants can be converted into hydrogels [15]. These CNT hydrogels can be simply prepared by heating a CNT dispersion and are synthesized using a different method from bucky gels, a gelatinous room-temperature ionic liquid that contains single-walled CNTs [16,17], CNT gels using gelling agents [18], and thixotropic CNT gels induced by high CNT concentration [19,20]. Furthermore, since our gel is physically crosslinked via π–π interactions [21], it exhibits reversible sol–gel transitions [15]. In addition, this CNT hydrogel exhibits electrical properties derived from the incorporated CNTs. In previous studies, only four types of hydrogels following this principle have been discovered, consisting of two types of CNTs and two types of dispersants. Specifically, two types of single-walled CNTs ((6,5)-chirality CNT and CG300) with diameters of approximately 0.8 nm and two types of dispersants (C.I. Reactive Blue 21 and Cyanocobalamin (vitamin B_12_)) (Figure 1). In addition, geometrical considerations of the gelation mechanism revealed that it is highly dependent on the size balance between CNTs (diameter) and dispersants (core size) [15]. In the CNT dispersion, the hydrophilic dispersant is assumed to be anchored on the CNT surface, following the curvature of the CNTs. When the dispersion is heated, the dispersant undergoes repeated adsorption and desorption from the CNT surface, during which a portion of the CNTs are presumed to be crosslinked, similar to a wooden structure for building a log cabin. The reason why the feasibility of gelation strongly depends on the relationship between the CNT diameter and the size of the dispersant is presumed to be due to this crosslinking structure.

In this study, the following two new points regarding our CNT hydrogel will be examined. The first is to find new combinations of CNTs and dispersants that can result in hydrogels following this approach. We predict that gelation in this method requires an appropriate size relationship between the diameter of the CNTs and the size of the dispersant. To prove this hypothesis, our next interest was to find out whether there were other combinations of CNTs with diameters other than 0.8 nm and dispersants that would result in a hydrogel. We focused on tannic acid (TA), which, like C.I. Reactive Blue 21 studied in previous research, possesses a large polycyclic group and has a larger core size than phthalocyanine. We hypothesized that the use of TA as a dispersant would enable the gelation of CNTs with diameters larger than 0.8 nm and it was therefore investigated. The second is to explore potential applications utilizing the intrinsic properties of CNTs. Several related studies on tannic acid-based materials [22], thermoelectric generators [23], and conductive hydrogels [24] have been reported, providing a broader context for the present study. In addition, the CNT hydrogel mentioned above is expected to be applicable as coating materials, antistatic materials [16], and soft actuators [17]. In addition, CNT aerogels, obtained by replacing the solvent in CNT gels with air, are anticipated to have potential for thermoelectric (TE) power generation [25]. In this study, we particularly focus on the potential deployment of these materials for TE power generation.

TE power-generating devices are attracting attention as devices that can directly convert heat into electricity to cover the growing global demand for energy and to achieve a sustainable society. However, most conventional TE materials, such as Bi_2_Te_3_ [26] and PbTe [27], contain rare materials, raising concerns about cost and environmental impact [28]. By contrast, CNTs have recently been discovered to have thermoelectric conversion properties close to those of existing materials and have attracted attention as a new thermoelectric conversion material [29]. In addition, both p-type and n-type semiconducting properties can be realized in CNTs, enabling the construction of complete TE modules. In CNT-based TE devices, the use of porous three-dimensional (3D) structures such as CNT aerogels has been reported to enhance TE performance [30,31]. This study explores the TE properties of CNT hydrogels and aerogels as potential novel TE materials.

## 2. Materials and Methods

### 2.1. Preparing for New CNT Hydrogel

As mentioned in the introduction, our previous studies demonstrated that certain combinations of CNTs and dispersants can cause CNT dispersions to gel. Specifically, when the core diameter of the dispersant, which has planar molecules such as phthalocyanine, and the apparent diameter of the CNTs to which the dispersant is adsorbed on the surface match approximately, the CNT dispersion of that combination will form a hydrogel.

**Figure 2:**
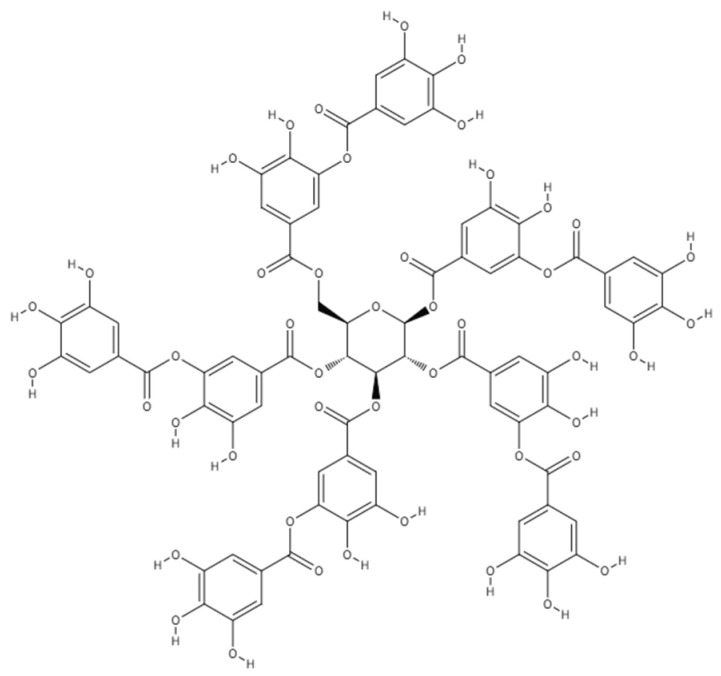
Chemical structural formula of tannic acid. Source: PubChem, CID 16129878. National Center for Biotechnology Information. https://pubchem.ncbi.nlm.nih.gov/compound/16129878 (accessed 9 July 2025).

In this study, TA (Figure 2) with a core diameter of 2.7 nm was found to be a dispersant [32] that could potentially induce hydrogelation of SG101-CNTs (single-walled, Zeon Corporation) with a diameter of 2–3 nm. Here, we focus on the gelation conditions of the prepared dispersion in relation to the gelation behavior and the concentrations of CNTs and TA. To investigate hydrogelation, a CNT dispersion was initially prepared using SG101-CNTs and TA as follows. Then, we verified whether the dispersion became a hydrogel.

1. Desired amounts of SG101-CNTs and TA are added to 10 mL of pure water in a vial.
2. While cooling the solution prepared in Step 1 in a cold water bath (4 °C), the CNT dispersion is prepared by using an ultrasonic homogenizer (UX-050, Mitsui Electric Co., Ltd., Chiba, Japan) for 2 h.
3. After Step 2, the dispersion is heated to 80 °C for 2 h.
4. After Step 3, the dispersion is assessed to see whether it gelled.

Here, to investigate the hydrogelation of CNT dispersions using SG101-CNTs and TA, CNT dispersions were prepared by varying the CNT concentration to 0.03, 0.05, 0.07, 0.10, 0.15, and 0.20 wt% while fixing the TA concentration to 0.15 wt% in the above Step 1, respectively. Additionally, CNT dispersions were prepared by fixing the CNT concentration at 0.05 wt% and varying the TA concentration to 0.05, 0.075, 0.10, 0.15, 0.175, and 0.20 wt%, respectively. Furthermore, we examined the gelation behavior using CNTs with different diameters, as well as under pH 3 and pH 11 conditions. The pH 3 condition was prepared by adding nitric acid (HNO_3_) to deionized water, whereas the pH 11 condition was prepared by adding potassium hydroxide (KOH) in deionized water. To elucidate the interaction between TA and CNTs during the gelation process, an attenuated total reflection Fourier transform infrared spectroscope (ATR-FTIR, FT-IR 6200, JASCO Co., Tokyo, Japan) equipped with a diamond crystal was employed for analysis.

### 2.2. Aerogelization of Proposed CNT Hydrogel

As mentioned above, we previously demonstrated that a new hydrogel could be obtained by combining SG101-CNTs and TA. However, including in our previous research, the potential applications of these hydrogels remained unexplored. One reason why the applied research using our hydrogel did not progress was that, in most cases, the hydrogel was so fragile that it could not be held by hand. As a solution, we developed a hydrogelation method for our hydrogel based on recently published research [33] that hydrogels can be obtained via hydrothermal treatment of single-walled CNTs and TA. This was inspired by the fact that the concentration balance of CNTs and TA is important for our hydrogel and that it is a heat-curable gel. The specific aerogel preparation method developed in this study is described below.

1. The CNT dispersion prepared in Section 2.1 is poured into a pressure-resistant container.
2. After Step 1, hydrothermal treatment is conducted on the hydrogel in the container in an autoclave at 160 °C for 24 h.
3. After Step 2, the hydrogel is frozen in a freezer, then thawed and dried at room temperature.

### 2.3. Evaluation of Thermoelectric Properties of Developed CNT Hydrogel and Aerogel

**Figure 3:**
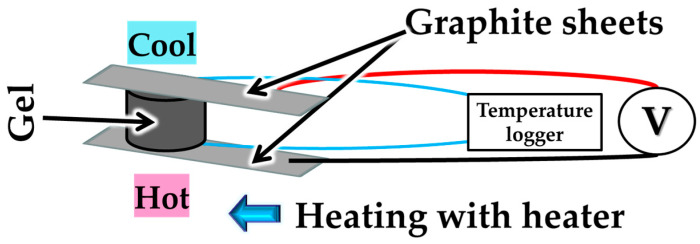
Measurement setup for CNT hydrogels and CNT aerogels.

Here, we considered the application of CNT hydrogels and CNT aerogels, which maintain a 3D structure, in thermoelectric conversion. Here, we investigated the thermoelectric conversion properties of each gel in the vertical direction. The measurement setup for the Seebeck coefficient is shown in Figure 3. As shown in the figure, electrodes are placed in contact with the bottom and top surfaces of the gel, and the bottom surface of the gel is heated by placing it in contact with a heater. (Room temperature will be on the relatively cooler side.) Graphite sheets are employed as electrodes during the measurement to prevent chemical reactions with the hydrogel. At this time, the temperatures of the bottom and top surfaces of the gel are measured, the temperature difference is recorded, and the electromotive force (E.M.F.) is measured and recorded at the same time. Measurements are conducted using a digital multimeter (IM TY720, Yokogawa Electric Co., Tokyo, Japan), a hot plate heater (SP88854200, Thermo Fisher Scientific, Waltham, MA, USA), and a temperature logger (LR5021, HIOKI E.E. Ltd., Nagano, Japan).

## 3. Results and Discussion

### 3.1. Relationship Between Gelation Behavior and Concentrations of CNTs and Tannic Acid

**Figure 4:**
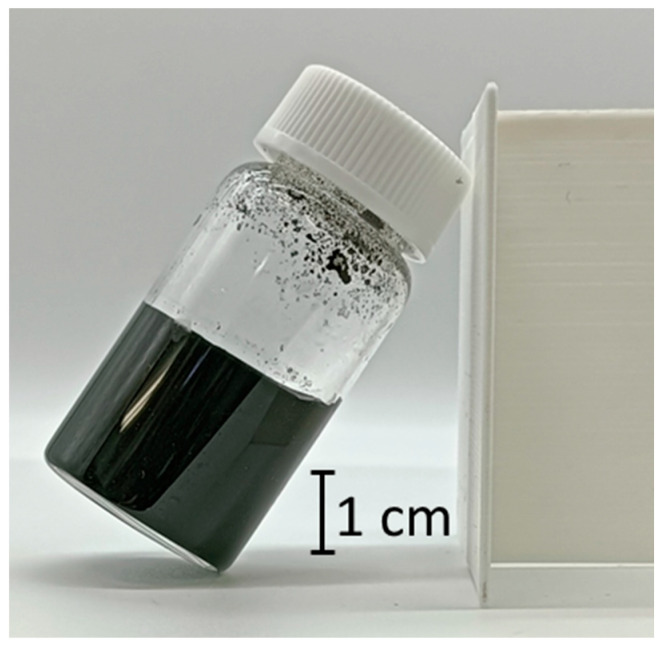
Discovered CNT hydrogel made from SG101-CNTs and TA.

In this study, we discovered that a CNT dispersion made from SG101-CNTs and TA with a core diameter of 2.7 nm became a hydrogel, as shown in Figure 4, using the method described in Section 2.1. First, we investigated the gelation conditions for the new CNT hydrogel we discovered.

Table 1 and Table 2 show whether or not gelation of the CNT dispersion was possible depending on the CNT concentration or TA concentration. Table 1 shows the relationship between the concentration of SG101-CNTs and the occurrence of gelation. The results indicated that the threshold SG101-CNT concentration for gelation using SG101-CNTs and TA (0.15 wt%) was 0.05 wt%. This hydrogel was supported by a CNT-based framework; therefore, when the CNT concentration was lower than this threshold, it was considered that a three-dimensional network with sufficient strength for gelation could not be formed. On the other hand, it was also confirmed that gelation did not occur when the CNT concentration was higher than 0.20 wt%. This was considered to be due to insufficient dispersion of CNTs. Two possible reasons for the poor dispersion are proposed. First, at higher CNT concentrations, 0.15 wt% TA was not sufficient to achieve adequate dispersion. Second, the increase in CNT concentration led to higher viscosity of the dispersion [34].

**Figure 5:**
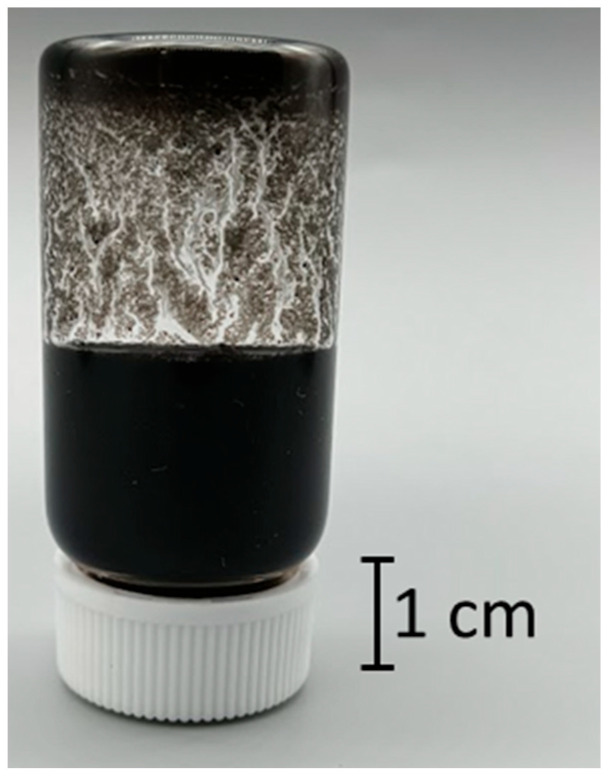
Incomplete CNT dispersion using SG101-CNTs (0.05 wt%) and tannic acid (0.05 wt%).

Table 2 shows the relationship between the concentration of TA and the occurrence of gelation. The results indicated that the appropriate concentration of TA for gelation with SG101-CNTs (0.05 wt%) and TA was 0.10 wt% to 0.15 wt%. When the concentration of TA was lower than this threshold (0.075 wt%), complete dispersion was not achieved, as shown in Figure 5, at the time of preparing the CNT dispersion prior to the gelation experiment. Partial gelation was observed when the concentration of TA was 0.75 wt% or 0.175 wt%. However, when the vial was tilted to a certain angle, the sample exhibited fluidity.

Furthermore, it was found that an excessive concentration of the dispersant TA inhibited gelation, preventing the formation of a hydrogel. This result was consistent with previous findings on hydrogels [15]. These findings suggested that the amount of dispersant must be carefully controlled within an optimal range.

### 3.2. Investigation of Gelation Feasibility by Combining Tannic Acid with CNTs of Different Diameters

In this section, we investigate the gelation conditions of CNT dispersions prepared with TA, focusing on the CNT diameter. Furthermore, we evaluate the validity of the results based on the crosslinking structure of the CNT hydrogel proposed in our previous study [15]. Here, we prepared four types of CNTs with different diameters, as described below, and investigated whether they could gel.

Table 3 shows the relationship between the diameter of CNTs and the occurrence of gelation. The result indicated that only SG101-CNTs gelled. CNTs with diameters either smaller or larger than that of SG101-CNTs did not form gels. Therefore, these findings suggested that gelation using TA in this method depended on the diameter of CNTs. Moreover, differences in the dispersion behavior were observed depending on whether the diameter of CNTs was smaller or larger than the appropriate diameter for gelation.

**Figure 6:**
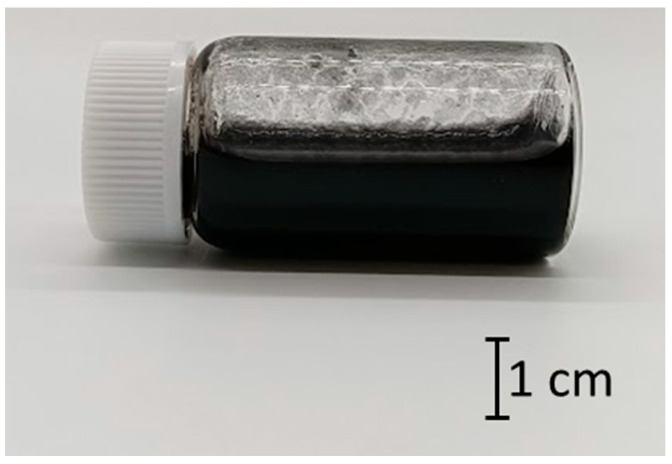
Photo of CNT dispersion using (6,5)-chirality CNTs.

**Figure 7:**
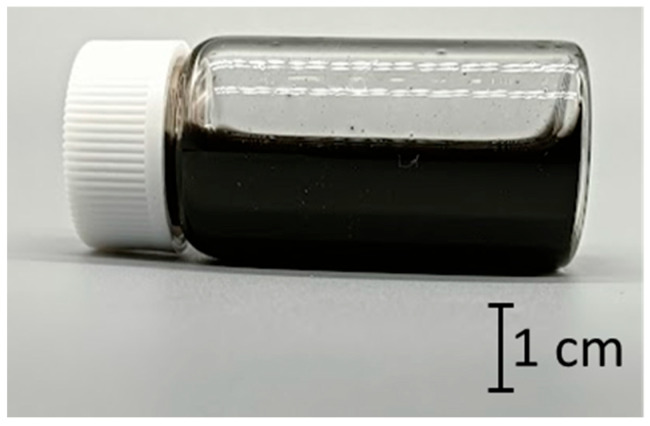
Photo of CNT dispersion using NC7000.

Figure 6 shows the result obtained from the sample using (6,5)-chirality CNTs, which had a smaller diameter than the appropriate diameter for gelation. Figure 7 shows the result obtained from the sample using NC7000, which had a larger diameter than the appropriate diameter for gelation. In the case of (6,5)-chirality CNTs, complete dispersion was not achieved. By contrast, NC7000 was successfully dispersed but failed to form a gel. These differences were likely attributable to variations in the number of TA molecules that could adsorb onto the CNT surface. The geometric considerations are as follows. It has been reported that pyrene derivatives, which are adsorbed onto CNT surfaces via π–π interactions, begin to desorb at around 50 °C [35]. In our previous studies, it was suggested that, upon heating, the CNT dispersion induced partial desorption of the dispersant from the CNT surface. As a result, the exposed hydrophobic CNTs aggregated in the water via hydrophobic interactions, thereby forming crosslinks [15]. Based on this hypothesis, a geometric model for the gelation of a CNT dispersion composed of SG101-CNTs and TA is presented below.

**Figure 8:**
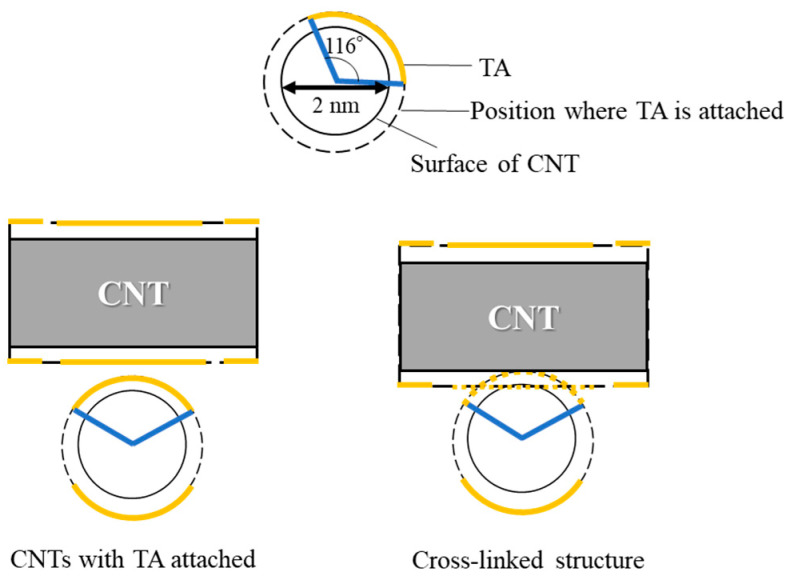
Size relationship with tannic acid when SG101-CNTs are used.

Figure 8 shows the estimated geometric relationship between SG101-CNTs and TA in the crosslinking structure of the CNT hydrogel. In this model, we assume that the diameter of SG101-CNTs is 2 nm and the maximum size of a TA molecule is 2.7 nm. The size of TA was determined based on a minimized 3D molecular model constructed via modeling software (MolView (https://molview.org)). In addition, the π–π interaction distance is 0.335 nm [36,37]. The distance of π–π interactions is generally considered to be equivalent to the interlayer spacing of graphite. When TA is adsorbed onto the curved surface of SG101-CNTs, it occupies approximately 116° of the SG101-CNT circumference. Accordingly, it is expected that three TA molecules can be adsorbed around the circumference of a SG101-CNT. When heat is applied to the dispersion, TA reversibly adsorbs and desorbs from the SG101-CNT surface, facilitating physical crosslinking between SG101-CNTs in a manner analogous to timber framing, as previously reported [15].

**Figure 9:**
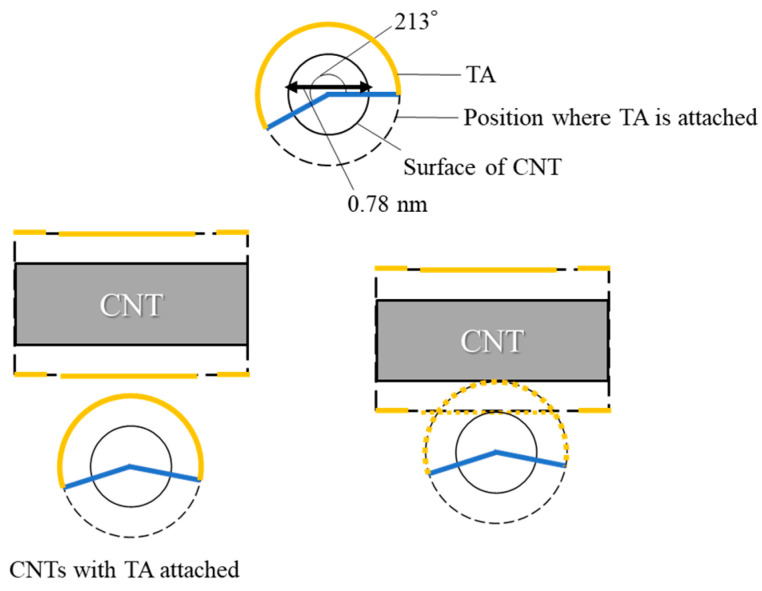
Size relationship with tannic acid when (6,5)-chirality CNTs are used.

**Figure 10:**
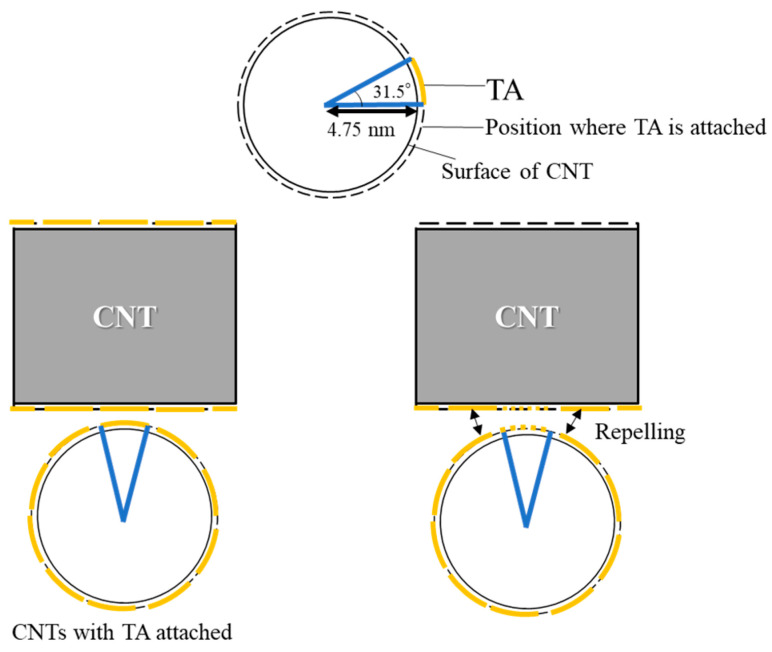
Size relationship with tannic acid when NC7000 is used.

Figure 9 and Figure 10 show the cases where the size relationship between CNTs and TA is not appropriate. When TA is similarly adsorbed onto (6,5)-chirality CNTs, it occupies about 210° of the circumference of (6,5)-chirality CNTs. Therefore, it is expected that only one TA molecule can be adsorbed around the circumference of these CNTs. When it is heated, if a single TA molecule is desorbed, the surface area of the exposed CNT increases, making it difficult to form a timber frame structure and increasing the likelihood of CNT aggregation. As a result, it is considered that a hydrogel will not be formed.

TA occupies about 30° of the circumference of NC7000. Therefore, it is expected that up to 12 TA molecules can be absorbed around the circumference of NC7000. Therefore, even if one TA molecule is desorbed from the CNTs, it is considered that crosslinking is prevented by repulsion by the other TA molecules. This trend is consistent with our previous study [15] and supports the proposed crosslinking structure suggested in earlier research. In gelation following this method, the previous study only confirmed that CNTs with a diameter too large for the dispersant do not gel. However, the results of this experiment confirmed that CNTs with a diameter too small for the dispersant do not gel either. These results strongly supported and substantiated the geometric considerations in previous studies that the formation of CNT hydrogels is dependent on the balance between CNT diameter and dispersant core diameter.

**Figure 11:**
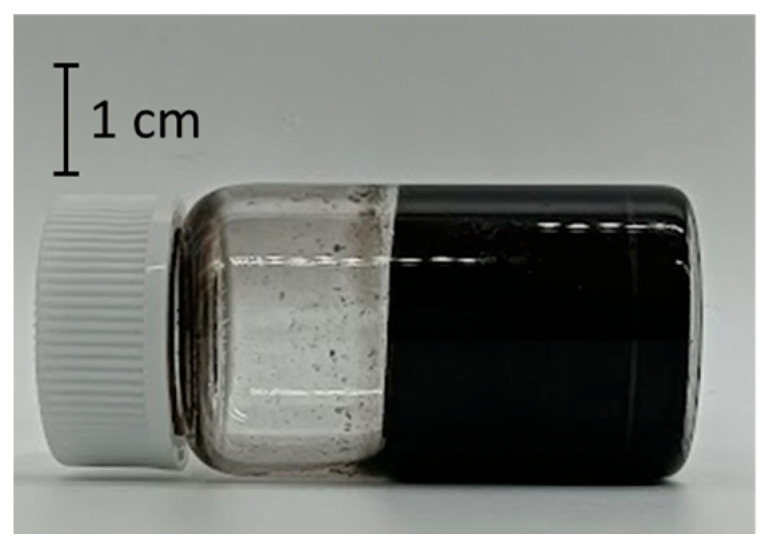
CNT hydrogel prepared using 7 mg of SG101-CNTs and 15 mg of tannic acid under pH 3.

**Figure 12:**
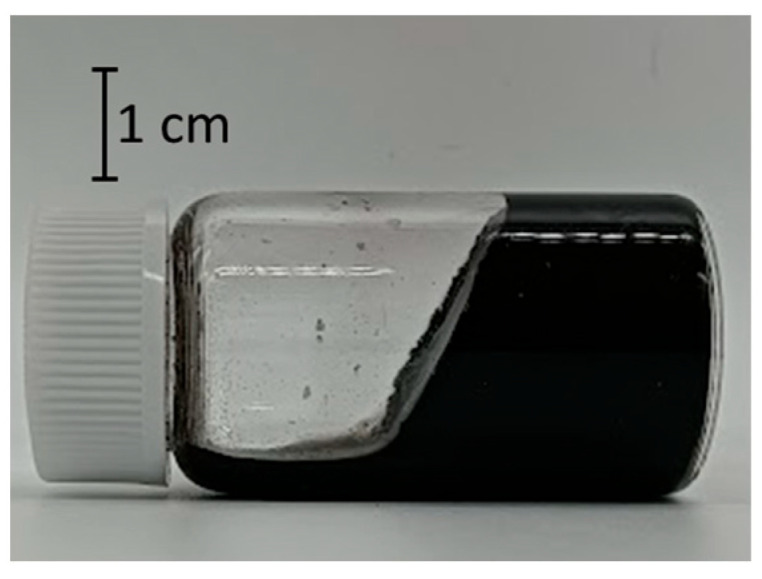
CNT hydrogel prepared using 7 mg of SG101-CNTs and 15 mg of tannic acid under pH 11.

Figure 11 and Figure 12 present the results of gelation conducted under pH 3 and pH 11 conditions. As shown in these figures, gel formation was observed under both conditions. Since TA is known to undergo decomposition under alkaline conditions [38], differences in chemical stability depending on pH may influence the mechanical strength of the gels. Furthermore, considering the absence of metal ions in acidic and neutral systems, it was reasonable to conclude that gelation was primarily governed not by ionic crosslinking but rather by noncovalent interactions, such as π–π stacking and hydrogen bonding.

**Figure 13:**
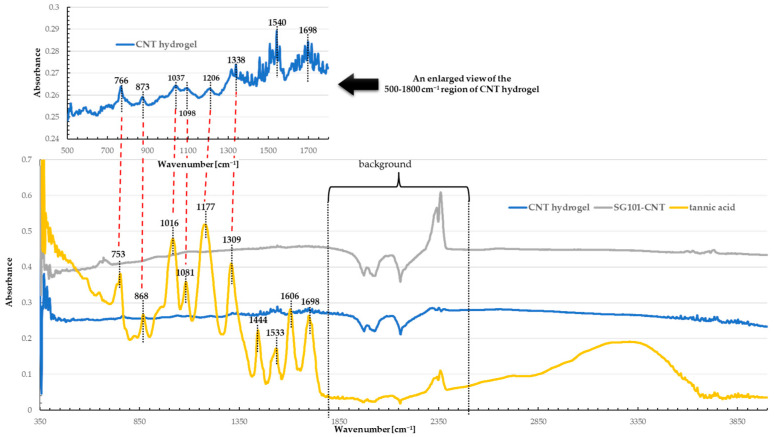
Infrared spectra of tannic acid, SG101-CNTs, and CNT hydrogel prepared using 7 mg of SG101-CNTs and 15 mg of tannic acid.

Figure 13 presents the infrared spectra of TA, SG101-CNTs, and CNT hydrogel prepared using 7 mg of SG101-CNTs and 15 mg of TA. Since a diamond crystal was employed as the ATR substrate in this study, the region between 1800 and 2500 cm^−1^ could not be measured. In the spectrum of TA, the peaks observed at 753–1309 cm^−1^ correspond to absorption bands arising from its characteristic chemical bonds. Moreover, the absorptions at 1444, 1533, and 1606 cm^−1^ are assigned to the stretching vibrations of the benzene ring [39]. As shown in Figure 13, the spectrum of the CNT hydrogel also exhibited peaks within the range of 766–1338 cm^−1^, which, despite shifts or attenuations, could still be attributed to those of TA. In addition, the peak at 1698 cm^−1^, assigned to the C=O stretching vibration of TA, was also observed at the same position in the CNT hydrogel without a noticeable shift. These results indicated that the interaction between CNTs and TA in the CNT hydrogel was predominantly noncovalent, involving π–π interactions and hydrogen bonding rather than covalent bonding [32,40].

### 3.3. Aerogelization of Proposed CNT Hydrogel for Its Applications

Here, CNT hydrogels were prepared using 7 mg of SG101-CNTs, 15 mg of TA, and 10 mL of pure water, following the procedure described in Section 2.1 and Section 2.2. The resulting gels showed differences in compressive fracture stress depending on the heating temperature: 4.4 × 10^2^ Pa at 80 °C and 4.4 × 10^3^ Pa at 160 °C. Furthermore, we discovered that when the CNT hydrogel was frozen and then dried at room temperature for 2–3 days, a sponge-like structure was formed (Table 4(c)).

The formation of an aerogel upon drying the CNT hydrogel was investigated with a focus on two factors: the heating temperature of the gel and whether it had been subjected to freezing. The results are shown in Table 4.

Heating at 160 °C likely enhanced π–π interactions for crosslinking [33], which helped the gel maintain its 3D structure during ambient drying. Furthermore, as shown in Table 4(c) and (d), when heated at 160 °C, the sample that was frozen became an aerogel, whereas the sample dried without freeze treatment lost its 3D network structure and became a sheet. Similar results have been reported that when preparing gels using carboxymethyl cellulose nanofibers and citric acid, conducting crosslinking under freezing conditions instead of at room temperature leads to an improvement in mechanical strength [41]. This is likely because freezing increases the density of reactive groups, which may promote the formation of multiple hydrogen-bonded clusters.

**Figure 14:**
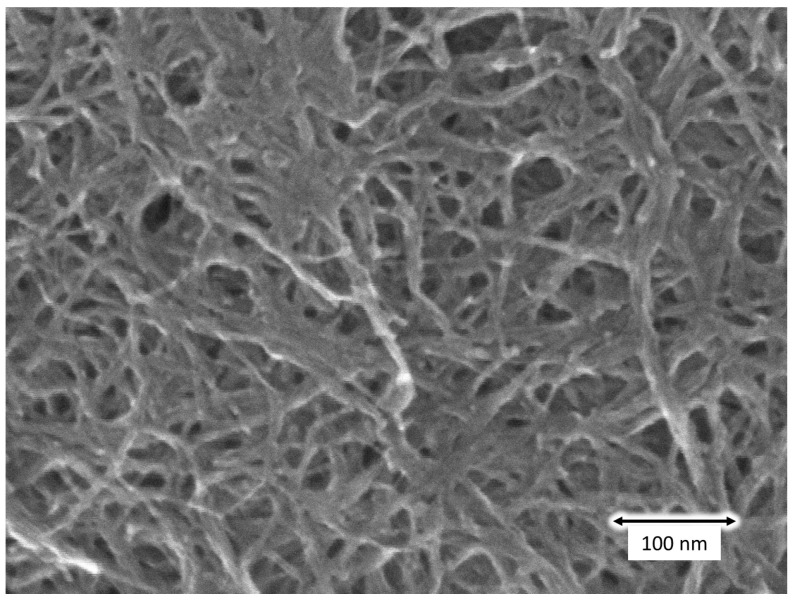
SEM image of obtained CNT aerogel using SG101-CNTs and tannic acid.

The microstructure of the prepared samples was evaluated using scanning electron microscopy (SEM, Hitachi High-Tech SU8010, Tokyo, Japan). Figure 14 presents an SEM image of the resulting CNT aerogel. Crosslinked and entangled CNT bundles were clearly observed. It has been reported that the pore sizes of CNT aerogels range from several nanometers to several hundred nanometers [42]. These SEM observations confirmed that our method enabled drying in ambient conditions, making it a simpler and more cost-effective approach.

### 3.4. Thermoelectric Performance of CNT Hydrogel and Aerogel

As mentioned above, we successfully obtained a 3D-structured CNT aerogel from the CNT hydrogel. Here, we considered the application of our gel as a TE conversion material. As explained in the introduction, CNTs are known to have excellent TE conversion properties. Although several studies have already been proposed the application of TE conversion materials combining CNTs with cellulose aerogels or other materials [25,43,44,45,46,47,48], our method is expected to be simpler than those methods. To evaluate their TE properties, the CNT hydrogel was prepared by following the procedure described in Section 2.1. The CNT aerogel was also prepared using the method described in Section 2.2. Then, we evaluated their TE conversion properties based on the electromotive force (E.M.F.) generated by the temperature difference using the method described in Section 2.3. The output E.M.F. was monitored for 20 min. Heating was initiated 30 s after the start of experiment and maintained at 100 °C for 120 s.

**Figure 15:**
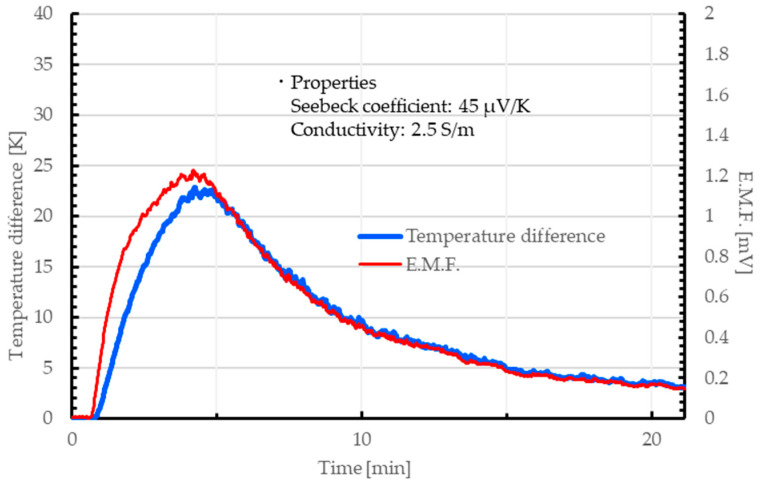
Relationship between temperature difference and E.M.F. in a CNT hydrogel composed of SG101-CNTs and tannic acid.

**Figure 16:**
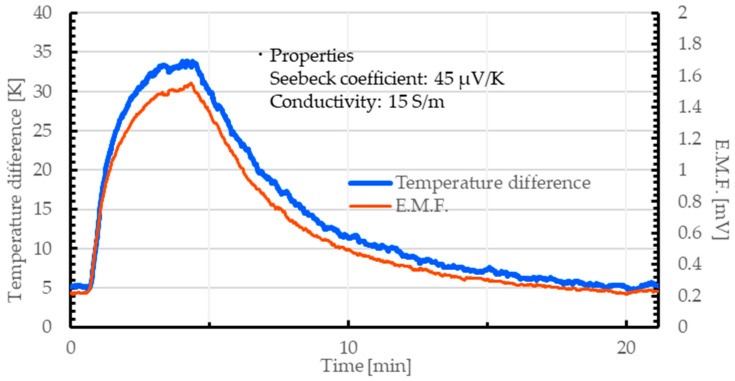
Relationship between temperature difference and E.M.F. in a CNT aerogel composed of SG101-CNTs and tannic acid.

Figure 15 and Figure 16 show the relationship between the applied temperature difference and E.M.F. obtained from the CNT hydrogel and aerogel, respectively. Both the CNT hydrogel and the aerogel exhibited stable TE output under the applied temperature gradient. In each measurement result, a TE conversion response that followed the applied temperature difference was observed.

The electrical conductivity and Seebeck coefficient of the CNT hydrogel were approximately 2.5 S/m and 45 µV/K, respectively. By contrast, the CNT aerogel showed values of about 15 S/m and 45 µV/K, respectively. These results demonstrated that both the CNT hydrogel and aerogel functioned as TE materials, exhibiting performance comparable to or even exceeding that of previously reported CNT-based thermoelectric materials [25,43,44,45,46,47,48]. In particular, our CNT aerogel was considered to have high feasibility in terms of TE conversion properties and strength.

## 4. Conclusions

We demonstrated the formation of a novel CNT hydrogel using SG101-CNTs and TA. Gelation was found to be strongly dependent on the concentrations of both CNTs and TA, as well as on CNT diameter, with successful formation observed for CNTs of 2–3 nm. Furthermore, by subjecting the hydrogel to hydrothermal treatment, followed by freezing and subsequent drying under ambient conditions, a CNT aerogel was obtained. Table 5 summarizes the properties of our CNT hydrogel and aerogel prepared using 7 mg of SG101-CNTs and 15 mg of TA. The mechanical strength of the CNT aerogel could not be accurately measured because its irregular shape and fragile structure made it incompatible with our laboratory-scale testing apparatus. Therefore, direct measurement using conventional methods was not performed. For the evaluation of thermoelectric figure of merit (ZT), measurement of thermal conductivity is necessary. However, since measuring thermal conductivity is difficult at this time, deriving ZT is challenging. We aim to clarify this in future research.

The Seebeck coefficients of the obtained CNT hydrogel and aerogel in this study were comparable to those reported for existing CNT-based TE materials. This result suggests that the facile fabrication of 3D TE materials from CNT dispersions could contribute to the development of next-generation energy-harvesting devices. It has been reported that the thermopower of mixed networks containing both semiconducting (s-CNTs) and metallic single-walled carbon nanotubes (m-CNTs) increases systematically with a higher concentration of s-CNTs in the network [40]. Therefore, the use of s-CNTs with well-defined diameters may enhance the Seebeck coefficient observed in this study.

## Figures and Tables

**Table 1:** Concentration of CNTs vs. gelation occurrence. (Concentration of TA is fixed at 0.15 wt%).

SG101-CNTs [wt%]	Gelation
0.03	Failure
0.05	Success
0.07	Success
0.10	Success
0.15	Success
0.20	Failure

**Table 2:** Concentration of tannic acid vs. gelation occurrence. (Concentration of SG101-CNTs is fixed at 0.05 wt%).

Tannic Acid [wt%]	Gelation
0.05	Failure
0.075	Partial
0.10	Success
0.15	Success
0.175	Partial
0.20	Failure

**Table 3:** Diameter of CNTs vs. gelation occurrence.

Types of CNTs	Length [µm]	Diameter (ave.) [nm]	Gelation
(6,5)-chirality CNTs (Single-walled)	1 (median)	0.78	Failure
SG101-CNTs (Single-walled)	300–500	2–3	Success
Double-walled CNTs	0.5–50	5	Failure
NC7000 (Multi-walled)	1.5 (average)	9.5	Failure

**Table 4:** Aerogel formation under various heating and freezing conditions.

		Pre-Drying Freezing	
		Conducted	No
Heating Temperature (sealed)	80 °C	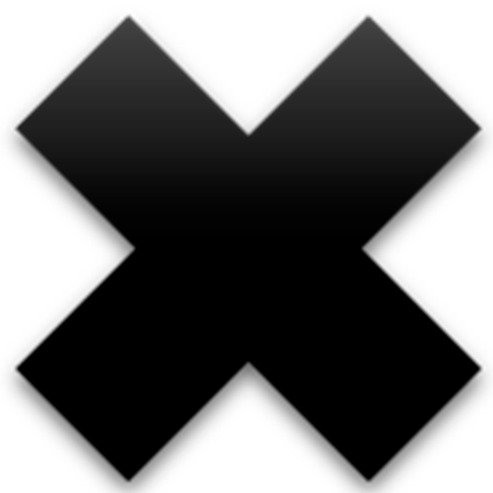 (a)	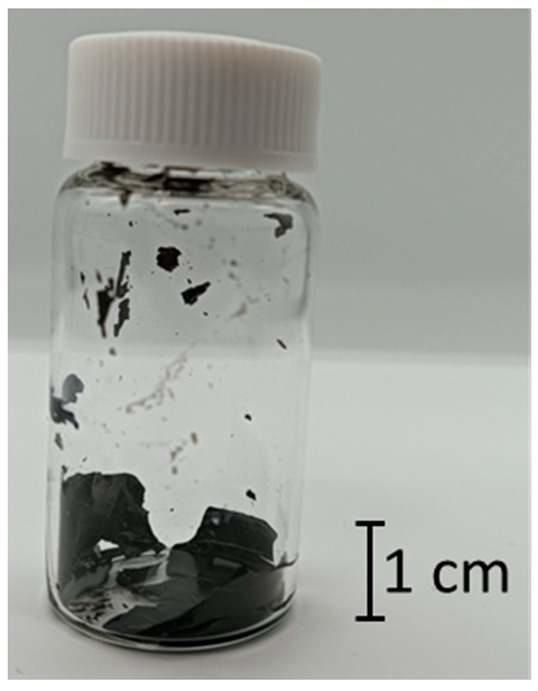 (b)
	160 °C	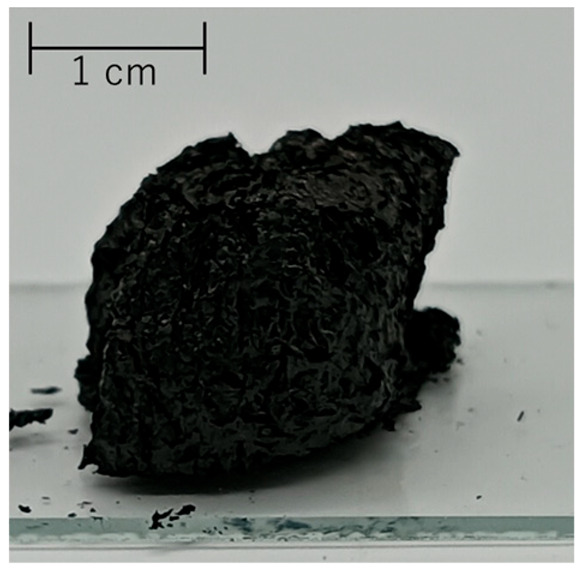 (c)	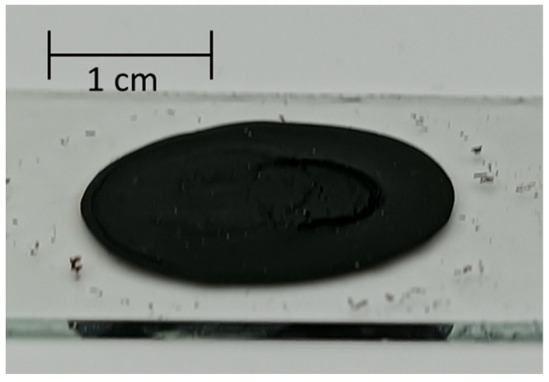 (d)

**Table 5:** Comparing the properties of our CNT hydrogel and aerogel.

	CNT Hydrogel (Heated at 80 °C)	CNT Hydrogel (Heated at 160 °C)	CNT Aerogel
Threshold concentration of CNTs (TA is 0.15 wt%)	0.05 wt%	0.07 wt%	N/A
Mechanical strength	4.4 × 10^2^ Pa	4.4 × 10^3^ Pa	N/A
Seebeck coefficient	N/A	45 µV/K	45 µV/K
Conductivity	N/A	2.5 S/m	15 S/m
Power factor (PF)	N/A	5.1 nW/(m·K^2^)	30 W/(m·K^2^)

## Data Availability

The original contributions presented in this study are included in the article. Further inquiries can be directed to the corresponding authors.

## Abbreviations

The following abbreviations are used in this manuscript:

CNT	Carbon nanotube
TA	Tannic acid
TE	Thermoelectric
3D	Three dimensional
E.M.F.	Electromotive force
PF	Power factor
ZT	Thermoelectric figure of merit

## References

[1] Ijima S. (1991). Helical micro-tubules of graphitic carbon. Nature.

[2] Popov V.N. (2004). Carbon nanotubes: Properties and application. Mater. Sci. Eng. R Rep..

[3] Odom T.W., Huang J.L., Kim P., Lieber C.M. (1998). Atomic structure and electronic properties of single-walled carbon nanotubes. Nature.

[4] Dresselhaus M.S., Dresselhaus G., Avouris P. (2000). Carbon Nanotubes: Synthesis, Structure, Properties, and Applications.

[5] Jorio A., Dresselhaus M.S., Dresselhaus G. (2008). Carbon Nanotubes: Advanced Topics in the Synthesis, Structure, Properties, and Applications.

[6] Gao C., Guo M., Liu Y., Zhang D., Gao F., Sun L., Li J., Chen X., Terrones M., Wang Y. (2023). Surface modification methods and mechanisms in carbon nanotubes dispersion. Carbon.

[7] Esbati A.H., Irani S. (2018). Effect of functionalized process and CNTs aggregation on fracture mechanism and mechanical properties of polymer nanocomposite. Mech. Mater..

[8] Zhu J.-M., Zare Y., Rhee K.Y. (2018). Analysis of the roles of interphase, waviness and agglomeration of CNT in the electrical conductivity and tensile modulus of polymer/CNT nanocomposites by theoretical approaches. Colloids Surf. A.

[9] Oya T., Ogino T. (2008). Production of electrically conductive paper by adding carbon nanotubes. Carbon.

[10] Yoshida M., Oya T. (2014). Development of carbon-nanotube composite thread and its application to “thread transistor”. Adv. Sci. Technol..

[11] Kamekawa Y., Arai K., Oya T. (2023). Development of transpiration-type thermoelectric power generating material using carbon-nanotube-composite papers with capillary action and heat of vaporization. Energies.

[12] Ampo T., Oya T. (2021). Development of paper actuator based on carbon-nanotube-composite paper. Molecules.

[13] Choudhary M., Sharma A., Raj S.A., Sultan M.T.H., Hui D., Shah A.U.M. (2022). Contemporary review on carbon nanotube (CNT) composites and their impact on multifarious applications. Nanotechnol. Rev..

[14] Kou Y., Oya T. (2023). Unique dye-sensitized solar cell using carbon nanotube composite-papers with gel electrolyte. J. Compos. Sci..

[15] Ogawa R., Arakaki R., Oya T. (2024). Development and geometrical considerations of unique conductive and reversible carbon-nanotube hydrogel without need for gelators. Gels.

[16] Fukushima T., Kosaka A., Ishimura Y., Yamamoto T., Takigawa T., Ishii N., Aida T. (2003). Molecular ordering of organic molten salts triggered by single-walled carbon nanotubes. Science.

[17] Fukushima T., Asaka K., Kosaka A., Aida T. (2005). Fully plastic actuator through layer-by-layer casting with ionic-liquid-based bucky gel. Angew. Chem. Int. Ed..

[18] Ogoshi T., Takashima Y., Yamaguchi H., Harada A. (2007). Chemically-responsive sol−gel transition of supramolecular single-walled carbon nanotubes hydrogel made by hybrids of SWNTs and cyclodextrins. J. Am. Chem. Soc..

[19] Shigeta M., Komatsu M., Nakashima N. (2006). Individual solubilization of single-walled carbon nanotubes using totally aromatic polyimide. Chem. Phys. Lett..

[20] Shaffer M.S.P., Windle A.H. (1999). Analogies between polymer solutions and carbon. Macromolecules.

[21] Nakashima N., Tomonari Y., Murakami H. (2002). Water-soluble single-walled carbon nanotubes via noncovalent sidewall-functionalization with a pyrene-carrying ammonium ion. Chem. Lett..

[22] Xiang H., Wang B., Zhong M., Liu W., Yu D., Wang Y., Tam K.C., Zhou G., Zhang Z. (2022). Sustainable and versatile superhydrophobic cellulose nanocrystals. ACS Sustain. Chem. Eng..

[23] Wei Q., Zhang Z., Sun W., Wu J., Wang X., Yu Y., Cai X., Zhang X., Wang L., Chen J. (2025). A versatile flexible film containing phase change microcapsules and polypyrrole/bacterial cellulose with high enthalpy, photothermal and Joule heating for personal thermal management. Chem. Eng. J..

[24] Yu Y., Ma T., Wei Q., Sun W., Tang J., Yu G., Xie W., Zhou G., Zhang Z. (2025). Highly Conductive, Stable, and Self-Healing MXene-Based Hydrogel Sensor via a Controlled Assembly of Polydopamine and Cellulose Nanocrystal. Energy Environ. Mater..

[25] Radouane N. (2023). Review on thermoelectric aerogels and their applications: Progress and challenges. J. Sol-Gel Sci. Technol..

[26] Pei J., Cai B., Zhuang H.-L., Li J.-F. (2020). Bi_2_Te_3_-based applied thermoelectric materials: Research advances and new challenges. Natl. Sci. Rev..

[27] Xiao Y., Zhao L.-D. (2018). Charge and phonon transport in PbTe-based thermoelectric materials. npj Quantum Mater..

[28] Hu X., Jood P., Ohta M., Kunii M., Nagase K., Nishiate H., Kanatzidis M.G., Yamamoto A. (2016). Power generation from nanostructured PbTe-based thermoelectrics: Comprehensive development from materials to modules. Energy Environ. Sci..

[29] Nakai Y., Honda K., Yanagi K., Kataura H., Kato T., Yamamoto T., Maniwa Y. (2014). Giant Seebeck coefficient in semiconducting single-wall carbon nanotube film. Appl. Phys. Express.

[30] Chen J., Gui X., Wang Z., Li Z., Xiang R., Wang K., Wu D., Xia X., Zhou Y., Wang Q. (2012). Superlow thermal conductivity 3D carbon nanotube network for thermoelectric applications. ACS Appl. Mater. Interfaces.

[31] Kang Y.H., Bae E.J., Lee M.-H., Han M., Kim B.J., Cho S.Y. (2022). Highly flexible and durable thermoelectric power generator using CNT/PDMS foam by rapid solvent evaporation. Small.

[32] Lin D., Xing B. (2008). Tannic acid adsorption and its role for stabilizing carbon nanotube suspensions. Environ. Sci. Technol..

[33] Liao W., Yu C., Peng Z., Xu F., Zhang Y., Zhong W. (2023). Ultrasensitive Mg^2+^-modulated carbon nanotube/tannic acid aerogels for high-performance wearable pressure sensors. ACS Sustain. Chem. Eng..

[34] Halelfadl S., Estellé P., Aladag B., Doner N., Maré T. (2013). Viscosity of carbon nanotubes water-based nanofluids: Influence of concentration and temperature. Int. J. Therm. Sci..

[35] Nakashima N., Okuzono S., Tomonari Y., Murakami H. (2004). Solubilization of carbon nanotubes with a pyrene-carrying polymer in water. Trans. Mater. Res. Soc. Jpn..

[36] Grimme S. (2008). Do special noncovalent π–π stacking interactions really exist?. Angew. Chem. Int. Ed..

[37] Pérez E.M., Martín N. (2015). π–π interactions in carbon nanostructures. Chem. Soc. Rev..

[38] Lin D., Liu N., Yang K., Zhu L., Xu Y., Xing B. (2009). The effect of ionic strength and pH on the stability of tannic acid-facilitated carbon nanotube suspensions. Carbon.

[39] Uematsu T., Suzuki M., Watanabe O. (2007). Structure of chemical coating of tannic acid on zinc plate studied by infrared spectroscopy and computational chemistry. J. Surf. Finish. Soc. Jpn..

[40] Zhao Z., Li L., Shao X., Liu X., Zhao S., Xie S., Xin Z. (2018). Tannic acid-assisted green fabrication of functionalized graphene towards its enhanced compatibility in NR nanocomposite. Polym. Test..

[41] Sekine Y., Nankawa T., Yunoki S., Sugita T., Nakagawa H., Yamada T. (2020). Eco-friendly carboxymethyl cellulose nanofiber hydrogels prepared via freeze cross-linking and their applications. ACS Appl. Polym. Mater..

[42] Hüsing N., Schubert U. (1998). Aerogels—Airy materials: Chemistry, structure, and properties. Angew. Chem. Int. Ed..

[43] Blackburn J.L., Ferguson A.J., Cho C., Grunlan J.C. (2018). Carbon-nanotube-based thermoelectric materials and devices. Adv. Mater..

[44] Li H., Zong Y., Ding Q., Han W., Li X. (2021). Paper-based thermoelectric generator based on multi-walled carbon nanotube/carboxylated nanocellulose. J. Power Sour..

[45] Abol-Fotouh D., Dörling B., Zapata-Arteaga O., Rodríguez-Martínez X., Gómez A., Reparaz J.S., Laromaine A., Roig A., Campoy-Quiles M. (2019). Farming thermoelectric paper. Energy Environ. Sci..

[46] Chen L., Lou J., Zong Y., Liu Z., Jiang Y., Han W. (2023). Wood-like aerogel for thermoelectric generators based on BC/PEDOT/SWCNT. Cellulose.

[47] Meng C., Liu C., Fan S. (2010). A promising approach to enhanced thermoelectric properties using carbon nanotube networks. Adv. Mater..

[48] Song H., Qiu Y., Wang Y., Cai K., Li D., Deng Y., He J. (2017). Polymer/carbon nanotube composite materials for flexible thermoelectric power generator. Compos. Sci. Technol..

